# Electrochemotherapy efficacy evaluation for treatment of locally advanced stage III cutaneous squamous cell carcinoma: a 22-cases retrospective analysis

**DOI:** 10.1186/s12967-017-1186-8

**Published:** 2017-04-26

**Authors:** Gianluca Di Monta, Corrado Caracò, Ester Simeone, Antonio Maria Grimaldi, Ugo Marone, Massimiliano Di Marzo, Vito Vanella, Lucia Festino, Marco Palla, Stefano Mori, Nicola Mozzillo, Paolo Antonio Ascierto

**Affiliations:** 1 Department of Surgery “Melanoma-Soft Tissues-Head & Neck-Skin Cancers”, Istituto Nazionale per lo Studio e la Cura dei Tumori “Fondazione G. Pascale”, Via Mariano Semmola, 80131 Naples, Italy; 2 Unit of Medical Oncology and Innovative Therapy, Istituto Nazionale per lo Studio e la Cura dei Tumori “Fondazione G. Pascale”, Via Mariano Semmola, 80131 Naples, Italy

**Keywords:** Electrochemotherapy, Electroporation, Squamous cell carcinoma

## Abstract

**Background:**

Extensive squamous cell carcinoma has few therapeutic options. In such cases, electrochemotherapy involving electroporation combined with antineoplastic drug appears to be a new potential option and may be considered as an alternative treatment. The aim of this retrospective single-center study was to evaluate electrochemotherapy efficacy in treatment of locally advanced stage III squamous cell carcinoma, in which surgical procedures would have entailed wide tissue sacrifice.

**Methods:**

Clinical features, treatment response, and adverse effects were evaluated in 22 patients treated with electrochemotherapy with intravenous injection of bleomycin for extensive stage III cutaneous squamous cell carcinoma. Treatment of cutaneous lesions were performed according to the European Standard Operating Procedures of Electrochemotherapy.

**Results:**

Overall response to electrochemotherapy treatment was observed in 18 (81.8%) patients. Clinical response with necrosis of tumor mass was observed from the first session and lasted for all follow up period that ranged between 5 and 48 months with a median of 34 months. Overall the treatment was well tolerated with a very low complication rate.

**Conclusions:**

Electrochemotherapy represents a safe and effective therapeutic approach, associated with a good tolerability.

## Background

Non melanoma skin cancers (NMSCs) are the most common type of skin tumor, representing about one-third of all malignancies diagnosed worldwide each year. Cutaneous squamous cell carcinoma (cSCC) is the second most common form of NMSCs and the risk of cSCC invasiveness should be assessed on the basis of tumor size, anatomical location, histological subtype and may result in nodal metastasis in 4% of cases. Patients with advanced inoperable skin tumors are frequently left with only few therapeutic alternatives. This subtype of giant cancer bears a higher risk of complication and mortality and, therefore, are also a great challenge considering surgical treatment. In such cases, electrochemotherapy (ECT) represents a potential treatment option. Electrochemotherapy is a recent therapeutic method used in primary and metastatic skin tumors. It is a safe procedure that can be considered especially when multiple lesions are present [1]. Application of high intensity electric pulses temporaly increase the permeability of cell membrane, thus allowing the direct diffusion of higly cytotoxic but not permeable molecule to entry within cells [2–6]. Among several drugs that have been evaluated in association to EP, bleomycin and cisplatin cytotoxicity was significantly augmented [5, 7, 8]. In addition to drug-induced cell killing, electroporation is responsible for changes in the tumor region. A “vascular lock,” consisting in a reflex constriction of vessels after electric pulse delivery, produces a temporary reduction in perfusion of tumor tissue and an interstitial edema [9, 10]. Furthermore, other vascular effects exerted by ECT include endothelial cell destruction and neovascular reorganization due to a local reduction in angiogenic factors production. In this retrospective study ECT efficacy and safety in treatment of locally advanced cSCC was evaluated.

## Methods

This was a retrospective, single-center analysis including 22 consecutive patients (7 females and 15 males) affected by cSSC, with median age at the diagnosis of 72 years (range 51–88) with extensive cSCC that were referred to the National Cancer Institute of Naples from January 2011 to December 2015. American Joint Committee on Cancer (AJCC) staging system based on objective criteria, was accounted to select patients. All patients included had T_2_ N_0_, M_0_ 7th edition cSCC tumor staging system characteristics, which is defined as tumor invading extradermal structures or more than 5 cm diameters, with no regional lymph node metastasis and no distant metastasis. Patient clinical characteristics are shown on Table 1. The histological characteristics of tumor were assessed. The technical procedure and patient selection were based on the ESOPE guidelines [11]. Inclusion criteria were: life expectancy longer than 6 months; measurable cutaneous or mucosal tumor lesions. Patients were offered ECT as a therapeutic option based on poor general condition, age, cardiac deficit not related to electrical malfunction, reduced lung performance, comorbidities, or that whether surgical procedure was deemed to be too invasive to be radical. Exclusion criteria included: clinically manifested arrhythmia, interstitial lung fibrosis, epilepsy, an active infection, a known allergy to bleomycin, kidney failure, previous treatment with bleomycin at the maximum cumulative dosage, and different anticancer therapies administered within 2 weeks of the ECT [8, 12]. Each patient was asked to give a written informed consent to participate to the study. Furthermore, demographic features including origin, age at onset, gender of the patient, as well as clinical features such as localization of lesions, treatment modalities, results and tumor recurrence at the time of observation were also recorded. This retrospective analysis was performed after the approval of an appropriate ethics committee (IEC of National Cancer Institute of Naples, reference number 44/09) in compliance with Helsinki Declaration. All patients underwent concurrent incisional biopsy for histological examination. All cases included in the study presented histopathologically confirmed cSCC lesions and underwent tumor staging by lymph node and abdominal ultrasound scan and chest X-ray as well as nuclear magnetic resonance (NMR) of tumor site for burden evaluation.

**Table 1:** Patients characteristics

No of patients	Sex	Age (years)	Localization	Tumor Characteristic	No of treatments	Duration of individual months follow up	Response
1	F	62	Nose	G_1_	1	22	CR
2	M	78	Scalp	G_1_	1	36	PR
3	M	75	Nose	G_1_	1	31	PR
4	M	72	Ear	G_2_	1	5	CR
5	M	64	Back	G_3_	1	36	PR
6	M	69	Forehead	G_1_	1	41	PR
7	M	53	Foot	G_3_	2	48	CR
8	M	54	Foot	G_2_	3	40	PD
9	M	73	Foot	G_2_	1	24	PR
10	F	51	Nose	G_1_	1	26	CR
11	M	64	Lip	G_2_	2	37	PR
12	M	87	Scalp	G_2_	2	40	SD
13	M	65	Foot	G_2_	1	26	CR
14	F	52	Cheek	G_1_	1	34	PR
15	F	84	Scalp	G_1_	1	26	SD
16	M	80	Ear	G_2_	1	28	PD
17	F	74	Cheek	G_1_	2	36	PR
18	M	88	Scalp	G_2_	1	31	PR
19	F	75	Scalp	G_1_	2	38	SD
20	M	78	Scalp	G_1_	2	35	PR
21	F	82	Nape	G_1_	1	34	PR
22	M	51	Leg	G_1_	1	32	PR

Overall response evaluated at 4 weeks is shown

### Electrochemotherapy treatment regimen

ECT treatment of cSCC lesions was performed according to the European Standard Operating procedures of Electrochemotherapy (ESOPE) [8, 11]. In 2006, the multicenter ESOPE project has defined and validated the standard operating procedures to safely and effectively treat patients with cutaneous and subcutaneous tumor nodules with ECT, thus providing the necessary guidelines for the use of ECT in clinical practice [8]. The primary endpoint of the study was to evaluate the efficacy of ECT in the treatment of cSCC. Treatment outcome was evaluated according to the Response Evaluation Criteria in Solid Tumors (RECIST-Guidelines) [13]. The term “complete response” means clearance lesions on later visits when compared with the first lesions on admission. “Partial response” equals at least 30% decrease in diameter of target lesions whereas “stable disease” involves less than 30% decrease. The terms complete and partial response as well as stable disease implicate the absence of new lesions or of progressive lesions. The electric pulse generator used in this study was the CE certified medical device Cliniporator_ (IGEAS.p.A., Carpi, Modena, Italy). The delivery devices were N-20-4B linear needle electrodes for head and neck lesions and N-20 HG needle electrodes for all other, which were inserted at subcutaneous level directly into deep tumor tissues and surrounding areas up to 2 cm of safe margin, so that the entire tumor tissue could lie within the electric field. Electric pulses were administered in a time interval of 8–28 min after intravenous injection of bleomycin at the dose of 15,000 IU/m^2^, in bolus, in a time interval of about 40–45 s, under general anesthesia. The patients were followed up at 1 and 4 weeks after the treatment and thereafter at monthly interval. The treatment was repeated in those patients who complete response was not achieved at a first ECT application.

### Assessment of response and toxicity

Clinical response was evaluated according to the Response Evaluation Criteria in Solid Tumors (RECIST). Adverse event were graded according to the National cancer Institute Common Toxicity Criteria 4.0. Measurement of lesions was performed before first treatment and at each follow up visit. Retreatment was given in case of stable disease or partially response up a maximum of 3 cycle of ECT.

## Results

**Fig. 1:**
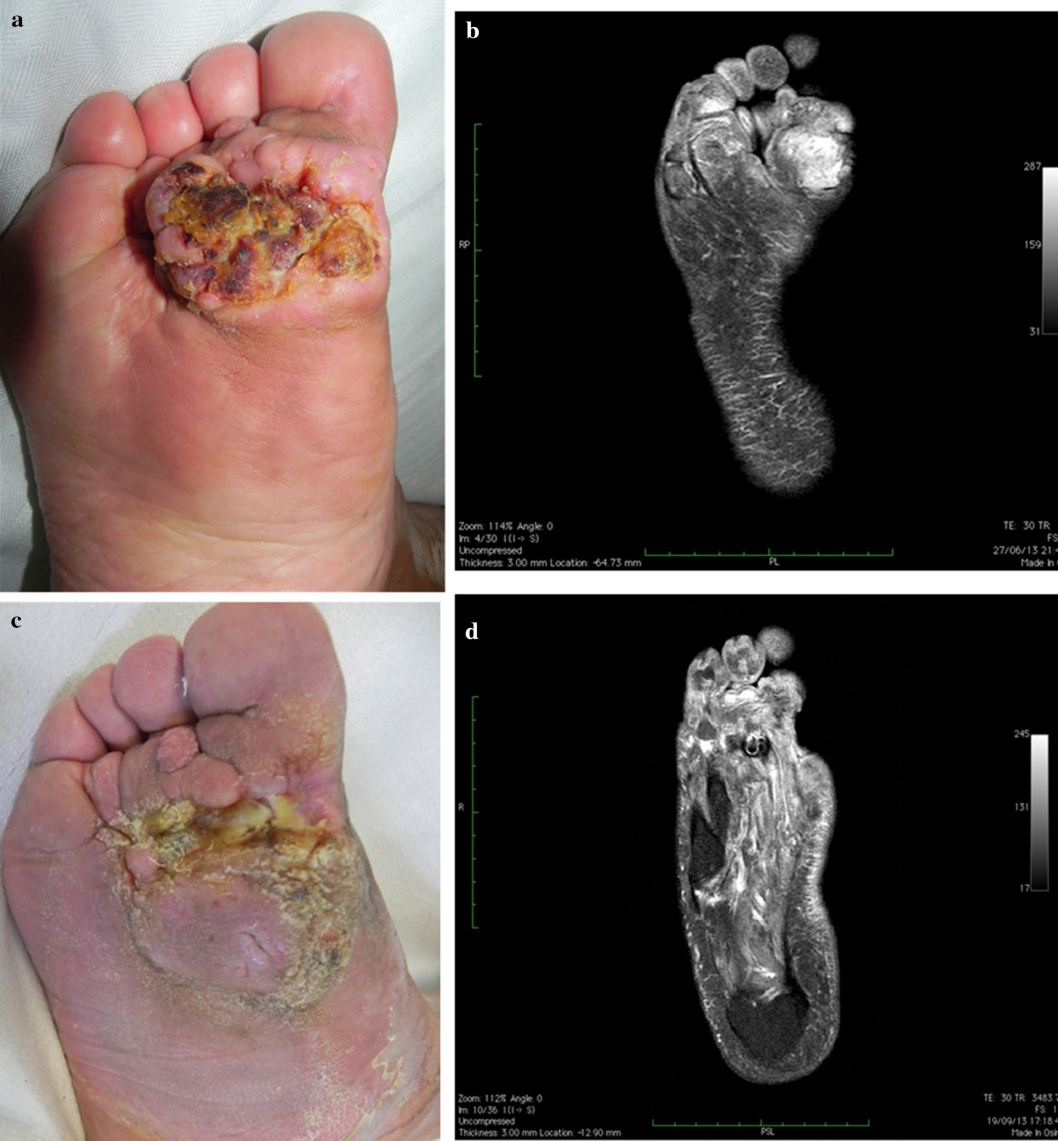
Advanced cSCC of forefoot. **a**, **b** Before treatment; **c**, **d** after 2 ECT sessions 12 weeks

**Fig. 2:**
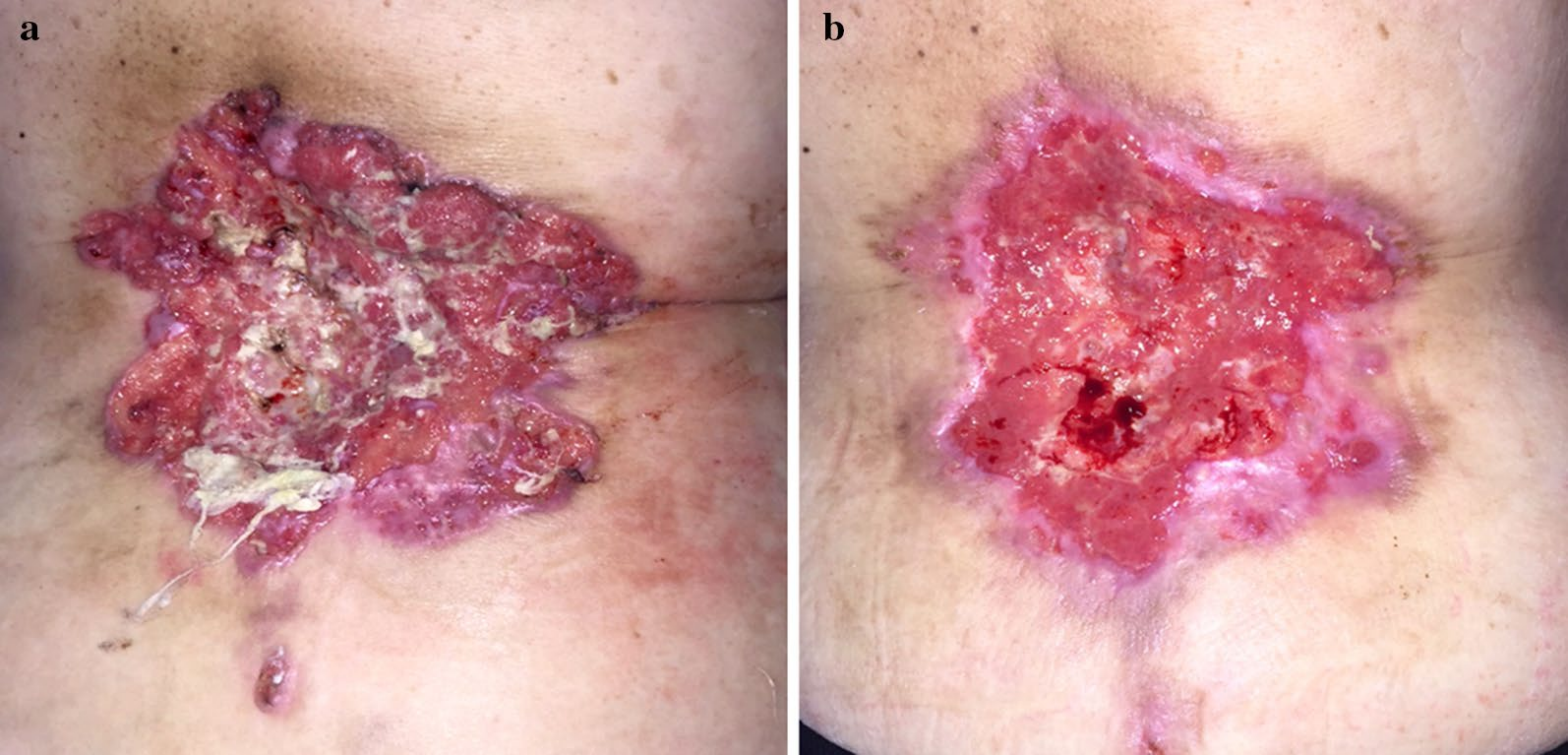
Advanced cSCC of back. **a** Before treatment; **b** after 1 ECT session 8 weeks

**Fig. 3:**
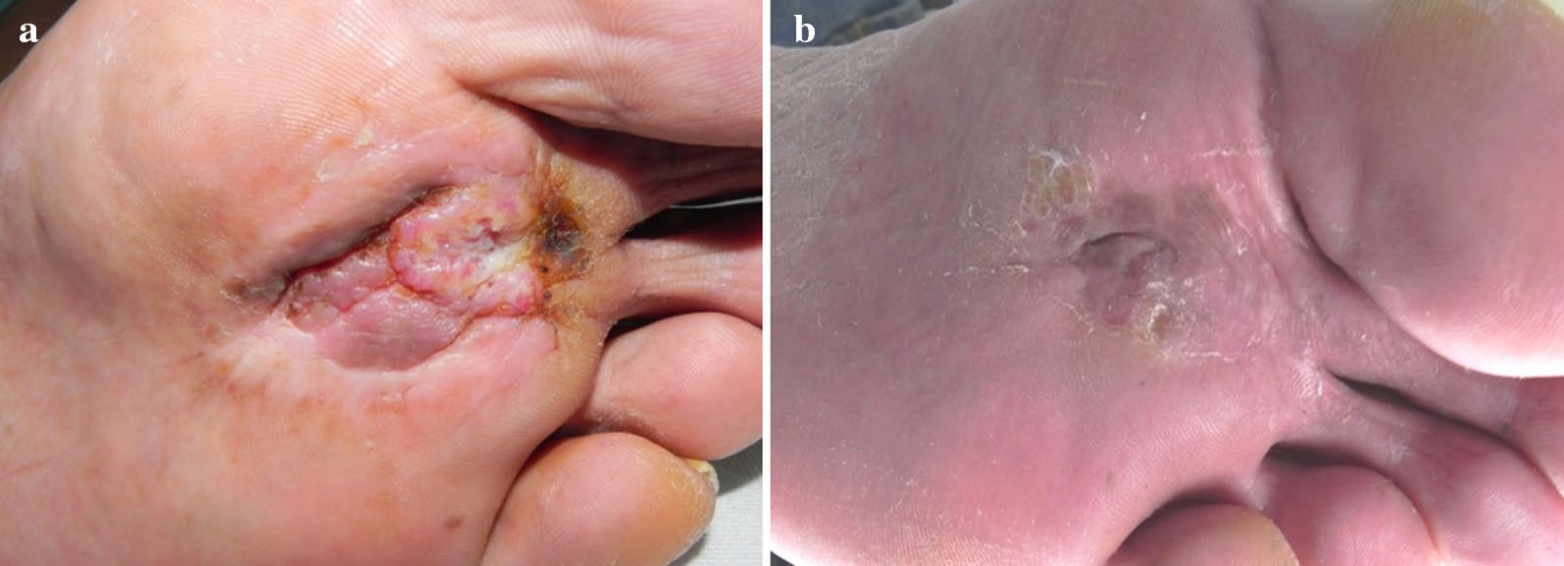
Advanced cSCC of forefoot. **a** Before treatment; **b** after 1 ECT session 8 weeks

According to RECIST guidelines an objective response (OR) to the first ECT treatment, scored at 4 weeks, was obtained in 18 (81.8%) of patients. Complete response (CR) was observed in 5 (22.7%) out of 22 patients, while in 13 (59%) cases a partial response (PR) was obtained and in 3 (13.6%) and 1 (4.5%) patients, respectively, stable disease (SD) and progressive disease (PD) were experienced (photo 1–3). Measurement of lesions were performed at each follow up visit. Patients with partial response or stable disease received a second cycle of ECT after 6 weeks for the first treatment and only 1 patient underwent a third cycle of treatment 8 weeks after. These results were observed during all follow up period that ranged between 5 and 48 months with a median of 34 months. The clinical response to ECT was evaluated 4 weeks after treatment and monitored every 3 months (Figs. 1, 2, 3). The patient who had progressed after 3 ECT sessions for a cSCC of the sole of his right foot was put out of the study and treated with amputation. All responsive patients showed a progressive improvement of pretreatment symptoms as local pain or functional limitation. Overall the treatment was well tolerated with a very low complication rate. Pain and erythema to the treated and surrounding area were among the most commonly reported side effects. Results are summarized on Table 1.

## Discussion

This study reports the results of a retrospective single center study aimed to the evaluation of the efficacy of ECT with intravenous bleomycin administration in stage III cSCC patients. Cutaneous squamous cell carcinoma is the second most common form of non-melanoma skin cancers and the risk of cSCC invasiveness should be assessed on the basis of tumor size, anatomical location, and histological subtype. Although most cSCCs are early diagnosed and successfully treated, in a small percentage of patients (4%) with AJCC stage III cSCC, lymph node metastasis may occur. When primary tumor surgical resection is not suitable, effective treatment options are limited to radio-chemotherapy. Radiation therapy is not an appropriate approach in advanced lesions invading bones, joints, or tendons. To date, there is no standard regimen treatment options for stage III cSCC and larger studies comparing therapeutic approaches are lacking. Chemotherapeutic agents showed a short-term response of 20–40% with polychemotherapy (cisplatin, 5-fluorouracil, taxanes, and gemcitabine). Even if the therapy of cSCC continues to evolve, at present the main goal of treatment in advanced SCC is control of local symptoms and prevention of symptomatic deterioration. In this scenario, the development of new therapeutic modalities with both palliative and curative potential it is greatly desirable. Quality of life and functional outcome play an increasing role in the different therapeutic options. ECT is described as an alternative to palliative chemo or radiotherapy and partial and complete remission rates have been reported in various clinical trials with low frequency of side-effects [7, 14–17]. Our experience confirmed the absence of major systemic side effects, with a good acceptance of ECT by all the treated patients. On the other hand, ECT may be the therapy of choice instead of salvage radical surgery, the survival rate of which does not exceed 25%. Electrochemotherapy is an effective treatment option for skin cancer, whatever histology to be treated, with high response rate achieved after the first procedure [18]. According to several authors, ECT treatment of skin cancer lesions on tumors with maximal diameter equal to or larger than 3 cm provides OR in 68.2% of cases, regardless the histotype [19].

Not homogeneous results of ECT response rate in cSCC treatment are described in previous papers (Table 2).

**Table 2:** Synoptic view of selected studies of ECT in cSCC

First author, year published^a^	Type of study	No pts	CR	PR (%)	OR (%)	Follow-up (months)	Drug
Belehradek M et al., Cancer 1993 [20]	Phase I/II	8	57	15	73		
Allegretti JP et al., Laryngosc, 2001 [16]	Phase I/II	14	51	36	87		
Burian M et al., Acta Otolaringol 2003 [21]	Phase II	12	83	17	100		
Bloom DC et al., Eur J Sur Oncol 2005 [22]	Phase II	54	25	32	57		
Matthiesen LW et al., 2011[23]	Phase II	3					BLM i.t.&i.v.
Landstrom FJ et al., Acta Otolaryngol, 2011 [24]		15				24	BLM i.t.
Skarlatos I et al., 2011 [25]	Prospective multicenter	14	9 (64.2)	4 (28.5)	13 (92.8)	Nd	BLM i.t.&i.v.
Gargiulo M et al., Ann Surg, 2012 [26]	Retrospective	13	8 (61.5)	5 (38.4)	13 (100)	18 (range 4–48)	BLM i.v.
Mevio N, et al., Tumori, 2012 [27]	Prospective	13	7 (53.8)	4 (38.4)	11 (84.6)	8 (range 2–20)	BLM i.v.
Benevento R et al., Surgery 2013 [28]	Prospective	8	5 (50%)	3 (30%)	8 (100)	3	BLM i.v.
Solari N et al., J Surg Oncol, 2014[29]	Prospective	5	Nd			6	BLM i.v.
Seccia V et al., Anticancer Res, 2014 [30]	Prospective	8	2 (25)	5 (62)	7 (87)	9 (range 3–12)	BLM i.v.
Campana LG et al., Br J Oral Maxillofac Surg, 2014 [31]	Retrospective	24	6 (25)	6 (25)	12 (50)	14 (3–82)	BLM i.t.&i.v.
Landstorm FJ et al., Acta Otolaryngol, 2015 [32]		4	4		4	24	BLM i.t.
Landstorm FJ et al., Acta Otolaryngol, 2015 [33]	Prospective	18	Nd		(100)	Median 58 months	BLM i.t.
Domanico R et al., Drug Des Devel Ther, 2015 [34]	Experimental	4	0	3 (75)	3 (75)	1	BLM i.v.
Campana LG et al., EJSO, 2016 [18]	Prospective multicenter	35	14 (40.7%)	21 (60)	30 (85.2)	1–12 (evaluation at 2 months)	BLM i.v.&i.t., CDDP i.t.
Rotunno R et al., G It Derm Venereol, 2015[35]	Multicenter prospective not randomized phase II	25	13 (52)	7 (28)	20 (80)	13	BLM i.v.
Bertino G et al. Eur J Cancer, 2016 [36]	Prospective multi center	50	26/47 (55)	11/47 (24)	37/47 (79)	2	BLM i.t.&i.v.
Di Monta G et al., current study	Retrospective	22	22.7	59	81.7	34 (range 5–48)	BLM i.v.

^a^Some papers include also tumor of different histotype from SSCC

Burian et al. within the framework of a European trial published a study in which 12 patients were treated with ECT and a complete response rate of 83% was observed [21]. In an subsequent open-label, multicenter, phase II studies on 54 patients, 57% percent of response (31.4% of complete response and 40.7% of partial response) was reported [21]. Other authors refer about ECT response rate in skin cancer treatment including different histotype lesions (melanoma, basal cell carcinoma, SCC) ranging from 89.5 to 60% [18]. A response rate in line with these results was obtained by Gargiulo et al. in a study in which 25 patients with non-melanoma head and neck cancers (various histotype) were treated with ECT using bleomycin. Seventy-two percent of complete response and 28% of partial response was observed. According to Gargiulo et al., the role of ECT is not limited to palliative treatment, but may also be the definitive treatment in patients with inoperable head and neck cancer, especially in elderly patients. The Authors strongly supported the ECT employment as neoadjuvant therapy in those cases where first line surgical procedure would be too invasive to be radical [26]. Landstrom FJ in a recent paper describes the 5-year local tumor control, safety of treatment and survival after ECT, and the 1-year quality-of-life (QoL) was published. The tumor-specific 5-year survival was 75%. The QoL outcome 1 year after ECT showed a significant increase in problems with senses (taste, smell), speech, mouth opening and xerostomia [33].

Bertino et al. in a phase II clinical study (EURECA) collected data on patients with head and neck cancers. This study was the largest clinical trial of ECT on 150 patients with melanoma and non-melanoma skin cancers of the HN area. Fifty patients out 150 were affected by SCC. The study showed that small lesions had a higher response rate (≤3 cm OR 88%) whereas for tumors >3 cm in diameter OR was 68%. Primary tumours (CR 70%, PR 20%) responded better than secondary (recurrent/metastatic) tumours (CR 55%, PR 20%). In addition, tumours which were not treatment naive showed reduced effectiveness of. At 2 months from ECT SCC group showed the higher percentage of OR (79%) with 55% of CR and 24% of PR. Interestingly, for recurrent tumour nodules, previous surgery least affected the outcome compared to (chemo) radiotherapy or multiple treatments [36].

This retrospective, single-center study demonstrates OR after ECT treatment of stage III cSCC of 81% and CR of 22.7%. Actually, such discrepancy in ECT response rate must be ascribable to the lack of coherent tumor type patient’s collection of these foregoing studies. To date, no previous scientific report is available on cSCC stage III ECT efficacy in terms of clinical response. To authors’ opinion, higher CR rate of prior reports is the consequence of lower cSCC stage and less tumor malignant propensity cohort patients observed. Anyhow, ECT is confirmed to display more effectiveness of other therapeutic options in locally advanced cSCC treatment. For this reason, stage III cSCC not amenable for surgical resection, is more responsive to ECT than chemotherapy o radiotherapy alone. Furthermore, ECT shows lower morbidity and toxicity than other treatment options, thus resulting more tolerated by patients.

## Conclusions

Electrochemotherapy represents a favorable choice in term of effectiveness showing a high rate of OR rate achieved, avoiding demolitive surgery and providing oncological effect with good aesthetical and functional preservation. Furthermore, ECT is a simple, quick and easily manageable treatment in term of local and systemic toxicity allowing the treatment of patients not amenable to other therapeutic regimen.

## Abbreviations

ESOPE: European Standard Operating Procedures of Electrochemotherapy
NMSCs: non melanoma skin cancers
cSCC: cutaneous squamous cell carcinoma
ECT: electrochemotherapy
AJCC: American Joint Committee on Cancer
NMR: nuclear magnetic resonance
CR: complete response
PR: partial response
SD: stable disease
PD: progressive disease
